# Fault Seal Analysis in an Onshore Unconventional Gas Target, North Perth Basin

**DOI:** 10.1111/gwat.13026

**Published:** 2020-07-23

**Authors:** F. Mullen, R. Archer, G. Yielding, H. Boogaerdt

**Affiliations:** ^1^ The University of Auckland, Private Bag 92019 Auckland Mail Centre Auckland 1142 New Zealand; ^2^ Badleys Geoscience North Beck House/North Beck Lane Spilsby E23 5NB United Kingdom

## Abstract

During the 1980s, hydrocarbons were logged in aquifers during drilling of conventional gas wells in the Woodada gasfield. The gasfield is located in the North Perth Basin in Western Australia. Using Fault Seal Analysis Technology, our goal was to test the hypothesis that faults in the Kockatea Shale that are currently being reactivated may be leak prone. Wells proximal to faults with a fracture stability of greater than 5 MPa logged only methane. Wells proximal to faults with a fracture stability ≤5 MPa logged both methane and condensate in aquifers confirming that hydrocarbon leakage is correlated with critically stressed faults. This assessment assumes that fault rocks in the Kockatea Shale, which is a regional source rock and seal, comprise uncemented phyllosilicate rock. For the normal stress case, faults oriented west‐north‐west with moderate dip have the lowest integrity. For the strike slip stress case, faults oriented north‐west and west‐south‐west, with moderate to steep dip have the lowest integrity. If the Kockatea Shale fault rock is assumed to be a cemented phyllosilicate, then the fracture stability increases to 14 MPa for both the normal and strike slip case. In this case, Jurassic‐Permian fault intersections may be contributing to hydrocarbon leakage, however, this would require numerical modeling for confirmation. Based on leak off tests, the increase in pressure required to hydraulically fracture the formation varies between 10.7 and 13.8 MPa. The treatment pressures used during hydraulic fracturing may potentially exacerbate leakage in areas such as the Woodada gasfield.

## Introduction

Hydrocarbon production from unconventional sources is growing in Australia, accompanied by concerns about the hydraulic connectivity between shale gas formations and aquifers and the potential for contamination of aquifers during hydraulic fracturing. This paper evaluates the natural occurrence of hydrocarbons, in aquifers, overlying a conventional gasfield in Western Australia, whose source rock is a potential shale gas target.

In the United States, elevated concentrations of gas in aquifers were detected within a one kilometer radius of unconventional wells in north eastern Pennsylvania. Gas leakage was attributed to poorly constructed wells. However, changes in fault integrity associated with hydraulic fracturing could not be ruled out (Osborn et al. 2011). Groundwater impacts arising from the process of hydraulic fracturing were monitored in a 2‐year longitudinal study in the Marcellus Shale in the United States (Bart‐Naftilan et al. 2018). The authors attributed transient changes in water quality to drilling activity rather than to hydraulic fracturing. However, the integrity of faults overlying the gas target was not quantitatively assessed in either case. In the Timor Sea, Mildren et al. (2002) utilized fault analysis seal technology (FAST) and determined that critically stressed faults were correlated with oil slicks in the overlying Timor Sea indicating fault seal integrity needs to be locally assessed.

The concept of structural permeability created by fault slip is well documented (Mildren et al. 2002). Pressure‐driven continuous gas‐phase flow through unsaturated fractures in the source rock is possible (Etiope and Martinelli 2002).

A decrease in pressure in saturated faults may result in gas ex‐solving rapidly from solution, allowing gas to migrate upward through the fracture system (Cramer et al. 2002). Density‐driven buoyancy of gas microbubbles in aquifers is discussed by Etiope and Martinelli (2002).

**Figure 1:**
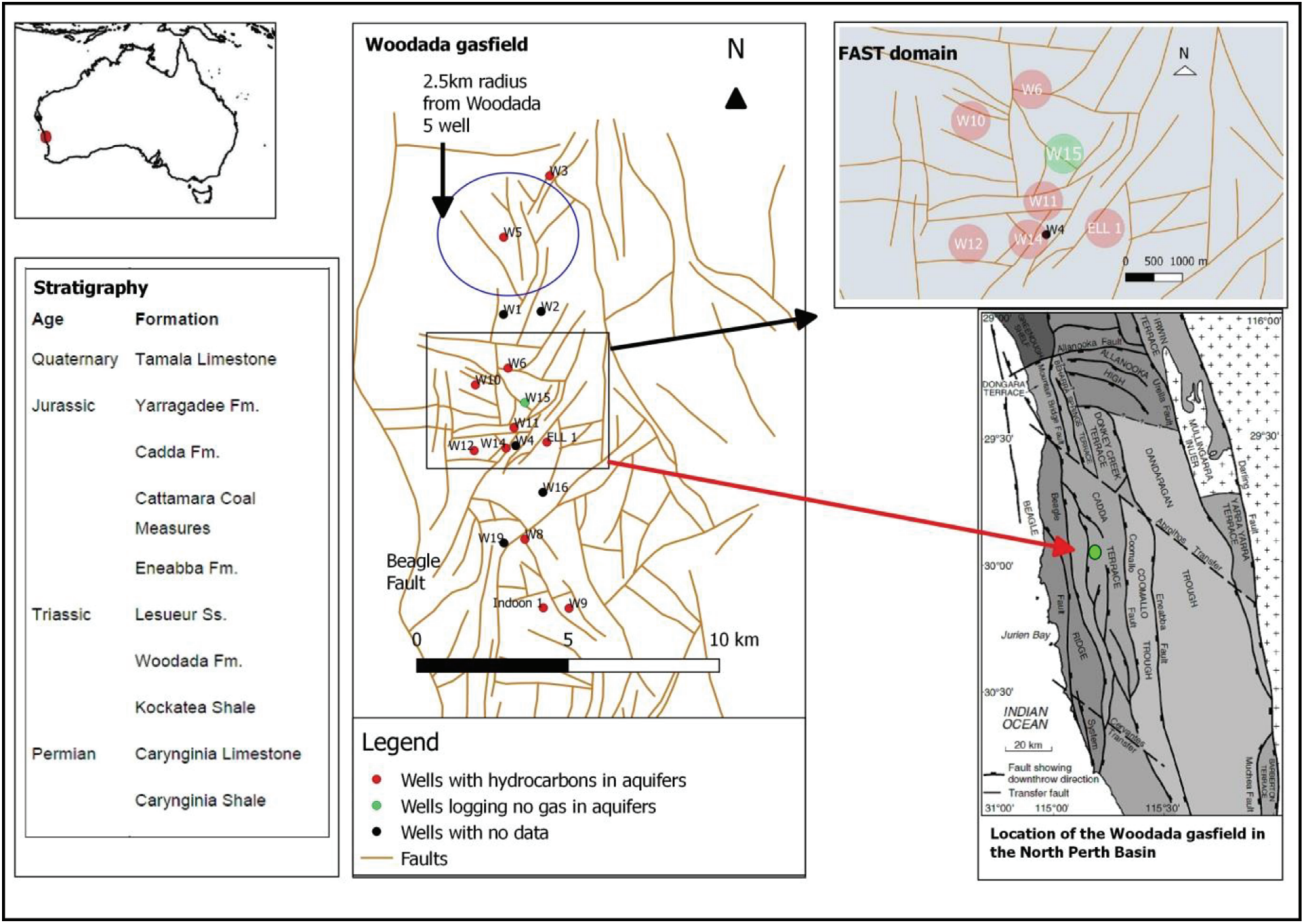
Center, top of the Carynginia Limestone fault map (Hydrocarbon Resource Group 1996). Top right, FAST domain, radii around wells indicate all wells are within 280 m of faults. ELL2 well is within 100 m of ELL1 and is not shown. Bottom right, tectonic elements of the onshore North Perth Basin (modified from Mory and Iasky 1996). Woodada gasfield is located on the Cadda Terrace (red arrow). Bottom left, stratigraphic table.

This study utilizes FAST to assess the source of hydrocarbons found in aquifers overlying the Woodada gasfield in south western Australia (Figure 1). The regional source rock (and seal) is the Kockatea Shale. It is known to be laterally extensive and continuous and is generally thick enough not to be completely offset by faulting (Crostella 1995). The underlying Carynginia Limestone was a conventional gas target in the 1980s and 1990s at Woodada gasfield. More recently the Kockatea Shale was a target for hydraulic fracturing (Norwest Energy unpublished data).

The following discussion focuses on possible drivers for leakage of hydrocarbons from the Kockatea Shale into overlying aquifers at the Woodada gasfield. The spatial distribution of hydrocarbons in aquifers across Woodada gasfield is considered in terms of both well integrity and fault integrity. FAST is employed to evaluate whether gas leakage correlates with critically stressed faults. Additional background information on the hydrogeological and geological setting is provided in Mullen et al. (2018), however, relevant background is also included herein.

## Geology

**Figure 2:**
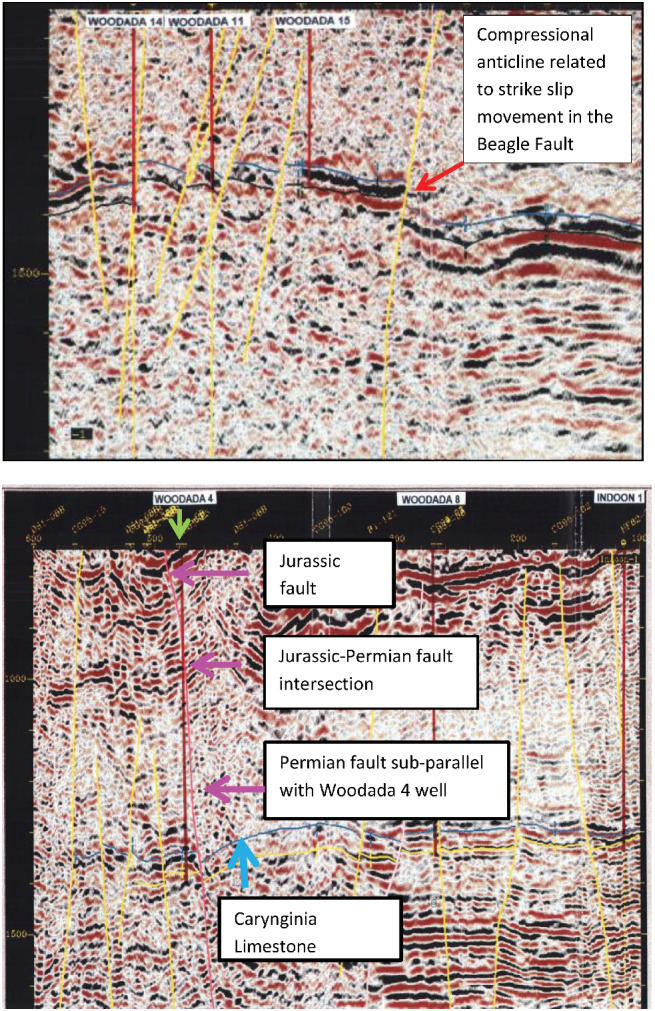
Top: south south‐west to north north‐east cross‐section of the Woodada anticline. Bottom: north–south cross‐section Woodada gasfield (seismic lines CG94‐16 and CG95‐08, respectively Hydrocarbon Resource Group 1996). The Carynginia Limestone is represented by the blue line. Reactivated Permian faults are colored yellow or red. Depth is represented in ms TWT.

The North Perth Basin formed through Permo‐Cretaceous rifting of Greater India and Australia (Figure 1). Oblique north‐west south‐east extension during rifting resulted in dextral strike slip movement along existing north striking faults such as the Beagle Fault. In locations where the Beagle Fault swings from northerly to north‐west and back to north north‐west, the hanging wall sequence is folded about its axis trending north‐west. This is consistent with compression in a restraining bend of the fault (Song and Cawood 2000). The Woodada anticline is interpreted to be an example of a compressional anticline related to strike slip movement in the Beagle Fault (Figure 2). Additionally, the Neocomian rifting event resulted in Permian faults extending upwards and Jurassic faults extending downwards (Figure 2). This appears to have been a regional phenomenon associated with continental breakup (Gartrell et al. 2004; Langhi et al. 2012).

## Hydrogeology

The stratigraphy of the area is presented in Figure 1. The shallow aquifers include the Yarragadee Aquifer and Cattamarra Coal Measures. The Yarragadee Aquifer has been eroded south of Woodada 14 well and only occurs in the north part of the Woodada gasfield. Groundwater salinity ranges from fresh at shallow depths to marginal near the base‐. Groundwater in the Cattamarra Coal Measures is low salinity in the southern half of Woodada gasfield (Commander 1978). There is no data for this aquifer in the northern half of the gasfield. Potentiometric data indicates the Cattamarra Coal Measures functions as a confining layer (Commander 1978).

The deep aquifers include the Eneabba Formation, Lesueur Aquifer, and Woodada Formation. In the Eneabba Formation, groundwater salinity is marginal in the southern half of Woodada gasfield based on water samples (Commander 1978). Groundwater in the Eneabba Formation and Lesueur Aquifer is brackish at Woodada 4 well based on resistivity log estimates (Mullen 2017). Groundwater is saline in both aquifers at the northern end of Woodada gasfield based on spontaneous log estimates . The increased salinity northwards is due to faulting and increased disconnection of aquifers across the Woodada anticline.

**Figure 3:**
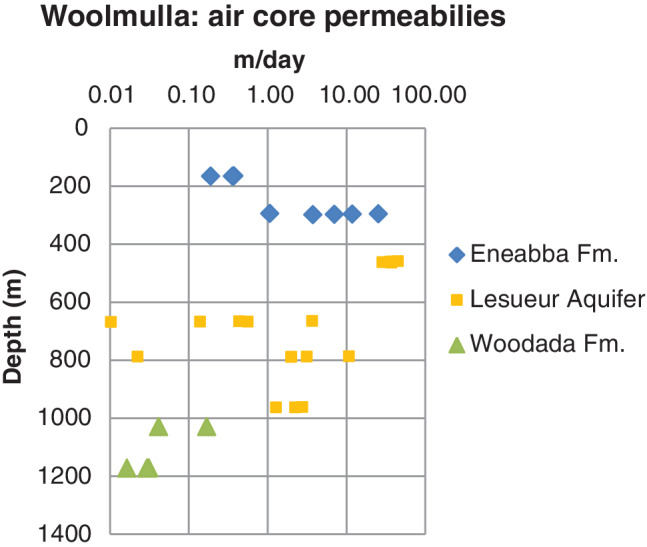
Core air permeabilities for the Eneabba Formation, Lesueur Aquifer, and Woodada Formation Woolmulla well.

While the Eneabba Formation and Lesueur Aquifer are hydraulically connected (Commander 1978), the Woodada Formation is hydraulically disconnected from the overlying aquifers (Mullen 2017). Returned water from the Woodada Formation during drilling of the Woodada 14 well indicated groundwater in the aquifer is saline (Consolidated Gas unpublished data). There is an order of magnitude reduction in the hydraulic conductivity of the Woodada Formation compared with overlying aquifers (Figure 3) however, the formation is assumed to be permeable to gas.

## Gas Data

Gas detection was based on the use of gas chromatography and hot wire detectors analyzing mud returns during drilling of new wells as reported in the well completion reports. While hot wire detectors are not a true reflection of the amount of gas in the formation, they can confirm whether gas is present in aquifers or not. An increase in mud pressure suppresses gas in the mud returns (Albard et al. 2012). Mud pressure was increased gradually with depth based on an examination of well completion report mud logs. Consequently, the impact of mud pressure on gas levels is assumed to be constant with depth. Gas was reported as total gas that includes gas spikes generated during changes in drilling activity. Gas chromatography can confirm whether the gas is thermogenic. Gas detection in the subsurface, based on the analysis of mud returns during drilling of new wells is discussed in Albard et al. (2012).

The Kockatea Shale produces a wet gas comprising a mixture of methane and condensate (short chain alkanes such as ethane, butane propane, and pentane). Six well completion reports provided the numeric record of gas levels in aquifers as well as a breakdown of the gas type. These include the Woodada 5, 6, 14, 15, ELL1, and the Indoon well. Written commentary in well completion reports indicate the type of gas found in aquifers during drilling and include Woodada 3, 9,10,11, 12, and ELL2 wells. Readers are referred to the Supporting Information for a summary of the gas data.

**Figure 4:**
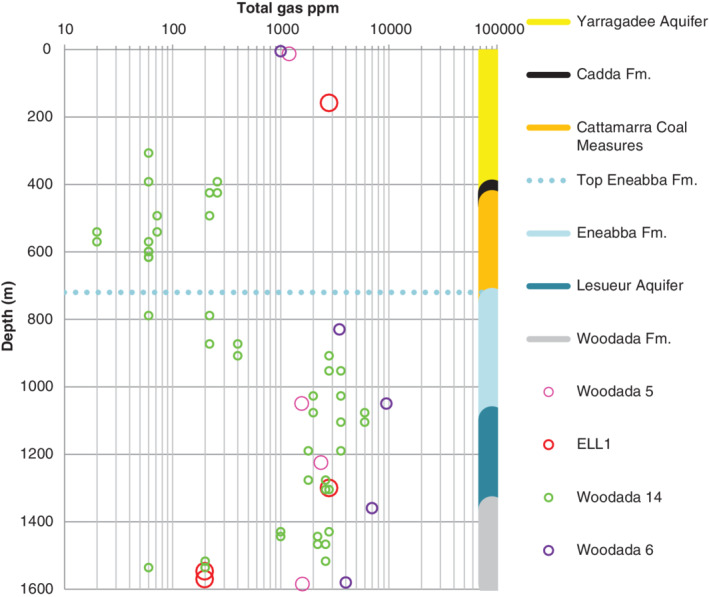
Average total gas in aquifers at the Woodada gasfield based on hot wire detection in mud returns. Note the *x* axis (gas levels) is a log scale. Stratigraphy on the right axis is referenced to the Woodada 14 well and consequently the gas data vs. depth is accurate to the level of the formation for the other wells. The data for the Indoon well is not shown due to its comparatively shallower stratigraphy relative to the main Woodada wells.

Total gas levels decreased at shallower depths in the Woodada 14, Woodada 6, and Woodada 5 wells (Figure 4). There was a noticeable reduction in the Eneabba Formation. Microbial degradation of hydrocarbons is optimal between 5 °C to 45 °C (USEPA 2017). Temperatures in the Eneabba Formation range from approximately 30 °C in the shallow part of the Eneabba Formation to 50 °C near its base (Commander 1978) suggesting biodegradation of hydrocarbons is possible at least in the upper part of the aquifer. The Eneabba Formation comprises red beds and these may provide iron as an electron acceptor (Fe^3+^ → Fe^2+^). Hydrocarbons can react with some Fe(III) minerals in an anaerobic aquifer matrix (Zachara et al. 2004). However, the rate of biodegradation is assumed to be slow compared to reactions with sulphate and nitrate (Appleyard 2020, personal communication).

Microbial oxidation of methane, primarily occurs in oxic environments and can consume a large proportion of methane (Bastviken 2009). If low salinity (well flushed) aquifers are assumed to be more oxic than saline aquifers (stagnant), then it can be inferred that low salinity areas may have relatively higher rates of hydrocarbon biodegradation. At Woodada 14 well (where the groundwater salinity is marginal to brackish), methane concentrations of 2102 ppm were logged in the basal part of the Eneabba Formation decreasing to 20 ppm in the upper part. At Woodada 5 well (where the aquifer is saline), methane concentration ranged from 1500 ppm in the basal part to 1200 ppm in the upper part of the aquifer. The contrast in the shallow gas concentration at the two locations suggests the possibility that aerobic biodegradation not anaerobic biodegradation (eg via Fe ^+3^) is having the greatest impact on hydrocarbon levels.

Methane was logged during drilling of most wells across the Woodada gasfield whereas condensate was logged in relatively fewer wells. In the FAST domain (Figure 1), methane only was logged at Woodada 10, ELL1, and ELL2 wells. Both methane and condensate were logged in aquifers at the Woodada 6, Woodada 11, 12, and Woodada 14 wells. There was no data for Woodada 4 well. No gas was logged at Woodada 15 well.

Whereas methane was found at all depths in most wells, there were some restrictions on the vertical distribution of condensate. Ethane was logged to the surface in some areas. The shallowest occurrence of propane and petroleum vapors was in in the Eneabba Formation. Butane was restricted to the Woodada Formation. The vertical stratification of condensate may reflect local variation in biodegradation rates as well as differences in mobility. The potential for preferential biodegradation of nonmethane alkanes compared with methane may account for their more restricted spread. However, this does not explain the complete absence of condensate in the vicinity of some wells.

If faults in the Kockatea Shale are leaking, higher gas readings would be anticipated in the Woodada Formation relative to the overlying aquifers. However, the opposite trend was observed in most wells. At Woodada 5 well, the gas levels decreased from 2360 ppm in the Lesueur Aquifer to 1590 ppm in the Woodada Formation. At the Woodada 6, well gas levels decreased from 10,100 to 8430 ppm and at Woodada 14, well gas levels decreased from 2800 to 60 ppm. No gas was logged in the Woodada Formation at ELL1 well. This was attributed to a temporary malfunction of the hot wire detector.

The Woodada Formation is hydraulically disconnected from the Lesueur Aquifer but it is assumed to be permeable to gas and if this is the case, gas could reach the Lesueur Aquifer relatively quickly even by matrix flow. However, the reduced gas concentration in the Woodada Formation suggests this may not be occurring to any significant degree. The Woodada 14 well crossed a fault in the Woodada Formation and the fault was producing water suggesting some faults in the formation may be permeable to water as well as gas. It is possible these may function as preferential flow paths connecting the Kockatea Shale to the more permeable Lesueur Aquifer. In this scenario, pressure gradients in the fault may result in hydrocarbons ex‐solving from solution resulting in their rapid migration via faults into the more permeable Lesueur Aquifer largely bypassing the Woodada Formation.

## Well Leaks

The Woodada gasfield is currently shut‐in on care and maintenance. Wells that are no longer in production are suspended prior to being decommissioned. Suspended wells are routinely pressure monitored on a six‐monthly basis until they are fully decommissioned. If monitoring results are not as expected, then further investigation is undertaken to determine if remedial action is required. In terms of well construction typically, a 13 3/8 inch casing was set in the lower part of Yarragadee Aquifer and cemented to the surface. A 9 5/8 inch casing was set in the Eneabba Formation or the Lesueur Aquifer and cemented to surface. A 7 inch liner was run to the basal Kockatea Shale or Carynginia Formation and this was cemented approximately 200 m above the casing shoe. A 4½ inch production liner was set in the Carynginia Formation. Cement was pressure tested and cement bond logs were used to confirm the top of the cement.

If well leaks are suspected, then wells postdating earlier wells would be expected to have higher concentration of gas logged during drilling. Woodada 5 well was the first well drilled with numeric records of gas levels provided in the well completion report. The gas logged during drilling of Woodada 5 well in 1982 is not easily attributable to leakage from previously drilled wells (Woodada 1, 2, 3, or 4) due to its relative isolation (Figure 1). The lateral spread of gas in aquifers was found to decrease significantly approximately 1 km from unconventional gas wells (Osborn et al. 2011).

Similarly the Indoon well drilled in1982 was the first well in the south part of Woodada gasfield and despite its isolation, methane was logged in relatively high concentrations in all aquifers . The nearest wells were drilled subsequent to the Indoon well. The Woodada 8 and 9 wells were both drilled in1984 and Woodada 19 was drilled in 2003.

The wells closest to Woodada 4 well (ELL1 and Woodada 14 wells) logged different hydrocarbons in aquifers. The ELL1 well is located 1 km east of Woodada 4 well and logged methane only. Woodada 4 well was drilled in 1981 and ELL1 well was drilled in 1983. Assuming methane is more mobile than condensate, methane detected in the ELL1 well could represent the early stage of a leak from Woodada 4 well.

Woodada 14 was drilled in 1995 and is 350 m west south west of Woodada 4 well. Unlike ELL1 well, the Woodada 14 well logged both condensate and methane. This may be due its closer proximity to Woodada 4 well relative to ELL1 and may reflect mobility differences between condensate and methane. Alternatively, it could represent a worsening leak at Woodada 4 well given Woodada 14 well postdates both the Woodada 4 and ELL2 wells by more than a decade. However, the evidence suggests Woodada 4 was probably not leaking during the production phase of the gasfield. The Woodada 4 well completion report indicated a satisfactory cement bond log at completion and low gas levels during drilling although no substantive detail was provided.

Woodada 5 was the only well with documented integrity issues during the production cycle of the gasfield. A poor cement bond was identified in the Carynginia Limestone above 2445 m. Pressure communication was identified between the 9 5/8 and 7 inch production casing annulus in the original well completion report . A surface pressure of 6.7 MPa was subsequently reported in both confirming pressure communication between the two liners (Australian Worldwide Exploration unpublished data). A surface pressure of 6.7 MPa equates to a pressure of 16.8 MPa at 1012 m in the Eneabba Formation (hydrostatic pressure of 10.1 MPa plus surface pressure). Leak off tests at this depth indicates the rock fractures at 16.6 MPa suggesting a high probability of gas leakage into aquifers at this location. The well required plug and abandonment due to the integrity issues identified in the original well completion report and was decommissioned by Australian Worldwide Exploration in 2014.

More recently Woodada 14 and Woodada 11 wells have required remedial interventions (Mitsui E&P unpublished data). Unlike Woodada 5 well, both wells had satisfactory cement bond logs and issues with well integrity were not apparent in the original well completion reports. However, the adjacent Woodada 4 well was deepened and hydraulically fractured in 2012 (Australian Worldwide Exploration unpublished data) and this may have subsequently impacted on the integrity of these wells. Woodada 14 well in particular was used for microseismic monitoring during hydraulic fracturing of the Carynginia Shale (Australian Worldwide Exploration unpublished data). It is assumed that issues with both wells significantly postdate the production phase of the gasfield.

## Fault Integrity

Structural issues that may be contributing to the elevated gas levels in aquifers across the Woodada anticline are reviewed. Langhi et al. (2012) found evidence for seal breach in optimally oriented faults in the offshore Kockatea Shale for a paleo‐stress field during rifting ie during the late Neocomian extensional regime. Additionally Jurassic‐Permian fault intersections created during the Neocomian rifting event have also been demonstrated to be leak prone in both compressional and extensional stress regimes based on numerical modeling (Gartrell et al. 2004; Langhi et al. 2012).

Similar risk factors occur in the onshore Kockatea Shale. Gas leakage from the Kockatea Shale was correlated with critically stressed faults for the current strike slip/normal stress regime across the Woodada anticline (Mullen et al. 2018). In addition, while linkages of Jurassic and Permian faults occur in the Kockatea Shale in the Woodada gasfield (Figure 2) no modeling has been conducted to determine the integrity of such intersections for the current stress regime.

**Figure 5:**
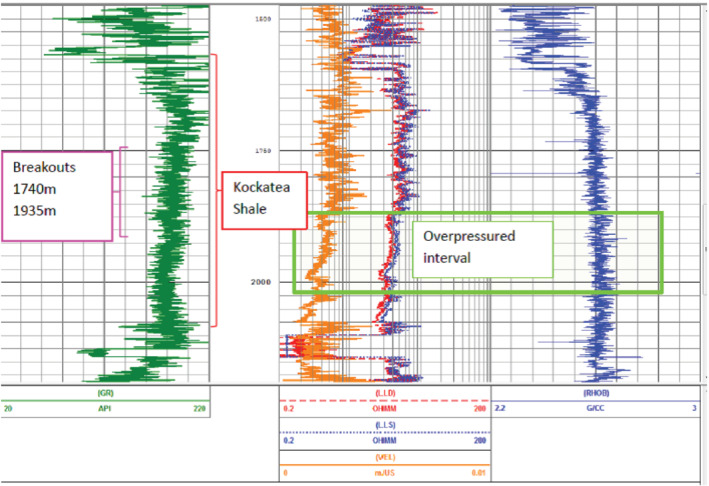
Geophysical logs from the Woodada 6 well shown in Data View (Schlumberger). Each small vertical increment is 25 m. Track 1: Gamma Ray log in green showing the uniform shale lithology of the Kockatea Shale. Track 2: Velocity log (VEL) orange, lateral log deep (LLD) in red, and the lateral log shallow (LLS) in blue. Track 3: the density log (RHOB) in blue. At approximately 1800 m, the velocity and resistivity logs (LLD and LLS) trend decreases and the density log (RHOB) trend shows a slight increase indicating a zone of overpressure which persists to 2000 m depth (green boxed area). The breakout interval is shown and discussed in the Methods.

Given the Kockatea Shale is in the oil window on the Cadda Terrace (Torghabeh et al. 2014) and is producing both gas and oil (Norwest unpublished data), hydrocarbon maturation may result in localized zones of overpressure and this may contribute to hydrocarbon leakage. While pore pressure in shale is difficult to measure accurately due to its low permeability, a decrease in velocity and resistivity and an increase in density in down hole geophysical logs can be used to provide a qualitative indication of pressured intervals (Bowers 2001).There is limited evidence of overpressure based on available geophysical logs in the Kockatea Shale for Woodada 6 well (Figure 5).

The Carynginia Limestone is a conventional gas target underlying the Kockatea Shale. Gas from the Carynginia Limestone has a similar composition to gas from the Kockatea Shale (Crostella 1995), and is therefore assumed to be sourced from the Kockatea Shale. Leakage of hydrocarbons via faults in the Kockatea Shale where it abuts the Carynginia Limestone indicates faults are permeable and that pressure is higher in the Kockatea Shale compared with the Carynginia Limestone. Static gradient tests in the Carynginia Limestone were not always reliable due to the complex dual permeability system in the reservoir . However, at Woodada 4 well, near the crest of the Woodada anticline, pressure gradients in the Carynginia Limestone were 0.0108 MPa/m compared with hydrostatic pressure gradient (0.0098 MPa/m). The mud pressure was also slightly above hydrostatic in the Kockatea Shale based on well completion reports. For example, mud pressure gradients in Woodada 6 well in the Kockatea Shale ranged from 0.0109 to 0.0112 MPa/m increasing with depth.

In summary, the distribution and type of gas logged in aquifers across Woodada gasfield is not easily reconciled with a well leak scenario as the only confirmed well with integrity issues during the production cycle of the gasfield was the relatively isolated Woodada 5 well. Hydrocarbons can potentially leak from crestal faults in the Kockatea Shale if those faults are optimally oriented in the current stress field and if there are localized zones of overpressure. Leakage is also possible where Permian and Jurassic faults intersect. Based on the gas logging data, if fault leakage is occurring hydrocarbon movement appears to be restricted to faults in the Woodada Formation with preferential flow occurring to the Lesueur Aquifer. The Lesueur Aquifer, which is significantly more permeable, is functioning as a gas collector zone.

## Method

**Figure 6:**
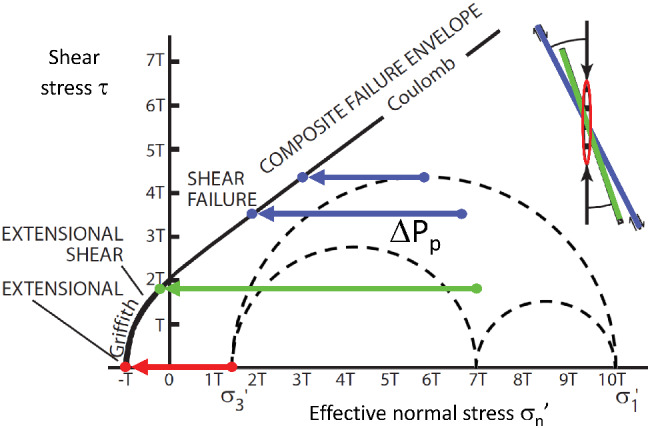
Three‐dimensional Mohr diagram with composite Griffith Coulomb failure envelope (Sibson 1996). The ΔP_*p*_ is change in pore pressure. *T* is tensile strength, *σ*′ is the effective stress (principal stress = *σ*′ + P_*p*_). The four colored points represent examples of the stress state on faults of different orientations. The blue arrows indicate ΔP_*p*_ which is the difference between the fluid pressure at the arrow heads at shear failure and the ambient stress at the tail of the arrows. The green arrow shows ΔP_*p*_ for extensional shear and the red arrow shows ΔP_*p*_ for extensional or brittle failure. The cohesion and coefficient of internal friction are defined by intercept and slope of the failure envelope.

Fracture stability is the increase in pore pressure required to force a fault into shear or extensional failure under conditions of Coulomb failure. The lower the calculated fracture stability (ΔP_*p*_), the higher the risk of fault plane reactivation and hence the higher potential for fluid leakage across the fault. The factors that affect the fracture stability are the stress state, the orientation and dip of the fault plane within the stress field, cohesive strength, and the coefficient of friction. The in situ tress tensor can be plotted on a Mohr diagram to determine the fracture stability of faults (Figure 6). A FAST map can then be produced by combining the reactivation risk stereonet with fault geometry and each fault segment is assigned a color coded ΔP_*p*_.

## Faulting

Seismic section CG95‐08 which crosses the Woodada 4 well (Figure 2) was assessed to determine if faults connect the Kockatea Shale to aquifers at that location. Depth data on the vertical scale is presented in time and covers an interval from 750 to 1600 milliseconds two way time (TWT). The top of the Carynginia Limestone is used as the reference point and occurs at 2133 m based on the Woodada 4 well completion report . This correlates with approximately 1350 ms suggesting a conversion of 1.6, that is, an average interval velocity of 3.2 km/s. The shallowest part of the cross‐section is at 750 ms which converts to 1200 m below ground. Four faults in the section project to depths shallower than 1200 m. At Woodada 4 well, the base of the Woodada Formation, which is the deepest aquifer, is 1435 m below ground confirming that the Kockatea Shale is connected to shallow strata by faulting.

Three seismic survey cross‐sections (CG98‐08, CG95‐15, and CG94‐16) including 26 faults were evaluated to determine true dip. The apparent dip was measured between the Carynginia Formation and the base of the Woodada Formation which is the deepest aquifer. Apparent dip in a vertically exaggerated cross‐section is related to true dip by:

(1) TanδE=Vtanδ

where δ*E* is apparent dip, δ is true, and *V* is vertical exaggeration. True dip ranged from 37° (for a single fault) to 88°, however, dip was generally between 70° and 80° (moderate to steep). In the FAST domain, Woodada 6, 11, 14, and 15 wells intercept faults at the level of the Carynginia Limestone. Woodada 10, 12, and ELL2 wells are within 280 m of faults at this depth as indicated from Figure 1. Proximity of wells to Permian faults at the level of the Woodada Formation is assumed to be approximately similar based on the relatively steep dip of Permian faults. Jurassic faults have reduced dip compared to Permian faults (Figure 2), however, fracture stability is not directly assessed for these faults.

## FAST Domain

**Figure 7:**
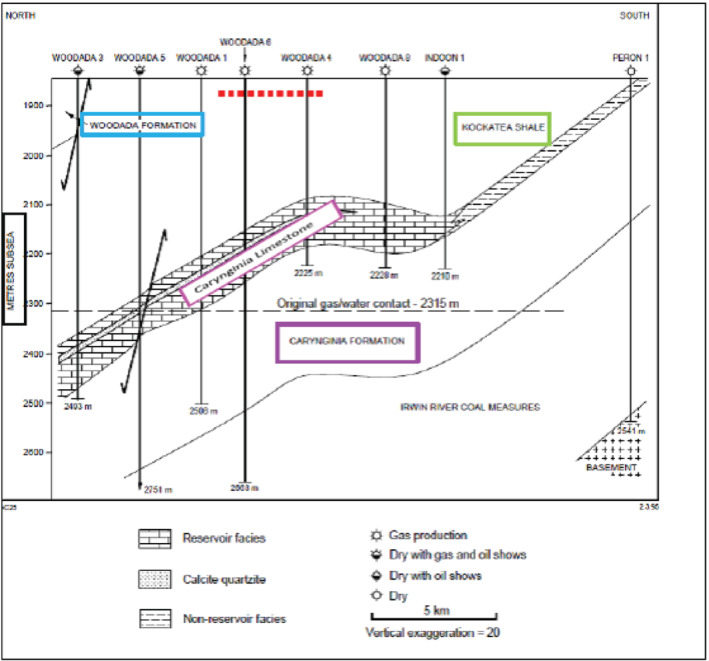
Schematic north to south cross‐section of the Woodada anticline in relation to the north dipping Cadda Terrace (modified from Crostella 1995). The red line indicates the FAST domain referenced to approximately 1910 m BGL or 1870 m subsea at the Woodada 4 well.

The three‐dimensional (3D) assessment of fracture stability is considered valid for the apex of the Woodada anticline and is based on Mullen et al. (2018) where stress data exists (Figures 1 and 7). Published breakouts (rated C) in the Kockatea Shale at the Woodada 4 well indicate an S_Hmax_ orientation of 110°N (Reynolds and Hillis 2000) which is consistent with the regional orientation of the S_Hmax_ from the same authors. The south part of the Woodada gasfield is excluded as low‐quality breakout data indicate a stress rotation with S_Hmax_ azimuth of 161°N at the Indoon well (Reynolds and Hillis 2000). A deviation from the regional S_Hmax_ will result in a likely change in associated tectonic strain and a screening level FAST assessment is not considered applicable in these circumstances.

## Rock Strength

Reliable fault strength data are required to undertake geomechanical fault reactivation analysis, however, there are no laboratory derived data for the Kockatea Shale in the Woodada gasfield area. Drill core data from other wells located on Cadda Terrace are used to provide some constraints on rock strength. Approximately 20 km to the south of Woodada gasfield at the Woolmulla well, the Kockatea Shale comprises 53% clay (Queensland University of Technology unpublished data). Approximately 15 km to the north, at the Arrowsmith 1 well, the formation comprises 30% clay (Queensland University of Technology unpublished data). Phyllosilicate fault rocks have clay volumes of between 15% and 40% and clay smears have clay >40% (Sperrevik et al. 2002). Woodada 4 well is approximately equidistant between the two wells on the Cadda Terrace and faults within the Kockatea Shale could potentially constitute phyllosilicate and/or clay smear.

Failure envelope inputs for unfaulted clay smear and phyllosilicate rock are shown in Table 1. These inputs were used in conjunction with Equation 2 (Nelson et al. 2006) to provide strength estimates.

(2) $$\mathrm{UCS}=2C/\left({\left(\mu^{2}+1\right)}^{0.5}-\mu \right)$$

**Table 1:** Failure Envelope Inputs for Fault Rock (Dewhurst and Jones 2003), Unconfined Compressive Strength is From Equation 2

	Coefficient Internal Friction	Cohesion (MPa)	UCS (MPa)
Clay smear	0.45	0.5	1.5
Phyllosilicate rock	0.6	0.5	1.8
Cemented phyllosilicate	0.85	10	43.2

where UCS is the unconfined compressive strength (MPa), *μ* = coefficient of internal friction (intact rock), and *C* = cohesion (MPa). Strength ranges from 1.5 MPa for a clay smear to 43 MPa for a cemented phyllosilicate. Note that faulted rock may be weaker or stronger than unfaulted rock (Dewhurst and Jones 2003) and these estimates are an approximate guide only.

The mineralogy from available drill core (Queensland University of Technology unpublished data) indicates an absence of smectite and the presence of kaolinite (7% at Woolmulla well and 1% at Arrowsmith 2 well). In addition, illite is a dominant component (11% Woolmulla well and 19% at Arrowsmith 2 well). Smectite is one of the weakest clay minerals whereas kaolinite and illite are significantly stronger (Dewhurst and Hennig 2003). Based on the clay mineralogy, a coefficient of friction of 0.4 is considered too low for the Kockatea Shale, however, it could reasonably range between 0.6 and 0.85. Consequently the faulted Kockatea Shale is assumed to comprise phyllosilicate fault rock and not clay smear (Table 1).

The Kockatea Shale occurs between 1572 and 2133 m at the Woodada 4 well. Breakouts occurred in the middle 35% of the formation between 1740 and 1935 m (Figure 5). A UCS of 32 MPa for the breakout interval was estimated using an empirical formula suitable for strong shale (Lashkaripour and Dusseault 1993):

(3) $$\mathrm{UCS}=1.001\;{ø}^{-1.142}$$

where ø is porosity derived from the Woodada 4 density log (Mullen et al. 2018).

Using Equation 2 and assuming the rock is a cemented phyllosilicate, reducing cohesion from 10 (from Table 1) to 7 reduces the UCS from 43 to 30 MPa which more closely approximates the log derived strength of the breakout interval.

The log‐derived rock strength above the breakout interval (1572 to 1740 m) was 22 MPa (Equation 3). Using Equation 2, reducing the coefficient of internal friction from 0.85 to 0.6 and cohesion from 10 to 5.8 MPa, the UCS was 20 MPa which approximates the log‐derived UCS for this interval. Woodada 4 well ran parallel with and close to a fault based on seismic data (Figure 2) and these strength estimates are assumed to reflect cemented phyllosilicate (Table 1).

The increased strength with increasing depth may be related to burial‐induced changes in the rock fabric. Where fault rocks have been buried to depths where temperatures exceed 90 °C, they can show enhanced pressure solution and quartz cementation which results in regeneration of mechanical strength (Dewhurst and Jones 2003). At Woodada gas field, the maximum burial depth of the basal Kockatea Shale is estimated to be 2858 m (Laker 2000). Using the current temperature gradient of 3.6 °C/100 m (Mory and Iasky 1996), temperatures in the Kockatea Shale at maximum burial would be ≥90 °C.

## Variation of Horizontal Stress with Depth

The occurrence of stress partitioning, which involves a change in the stress gradient with depth across different lithologies is likely to impact on the propagation of fault slip. The FAST assumes a constant stress gradient with depth and stress partitioning would invalidate the results. No leak off tests were conducted in the upper part of the Kockatea Shale or Woodada Formation. These would have provided some evidence of whether the S_hmin_ gradient changes with depth. Leak off tests in overlying deep aquifers indicate both increases (at the Woodada 5, Woodada 6, and Indoon wells) and decreases (at the Woodada 1 and ELL1 wells) of less than 2 MPa in the S_hmin_. However, these changes reflect the poro‐elastic characteristics of those formations and are not indicative of S_hmin_ in the upper Kockatea Shale or the Woodada Formation.

Horizontal stress can preferentially concentrate in strong rock and result in breakouts being restricted to those intervals. However, this degree of stress partitioning usually only becomes apparent when comparing significantly different lithologies (Nelson et al. 2006). As discussed previously, breakouts were concentrated in middle 35% of the Kockatea Shale between 1740 and 1935 m. The gamma ray log response in the upper part of the Kockatea Shale becomes less uniform compared with the breakout interval, however, the minimum API is still greater than 150 (Figure 5). The upper part of the Kockatea Shale still qualifies as shale. The relatively small decrease in rock strength observed above breakouts also suggests stress partitioning within the Kockatea Shale is unlikely.

Pore pressure is correlated with S_hmin_ (Dewhurst and Hennig 2003). There is evidence of minor overpressure in the lower half of the Kockatea Shale at Woodada 6 (Figure 5) and consequently the horizontal stress gradient may be relatively less in the upper part of the Kockatea Shale at least at this location. A relative decrease in the horizontal stress gradient in the upper part of the Kockatea Shale is assumed not to have an inhibitory effect on fault slip across the formation. Without definitive data, the stress gradient was assumed to be constant with depth in both the Kockatea Shale and Woodada Formation.

## Limits to Stress Using Frictional Fault Theory

On the crest of the Woodada anticline at 1910 m depth in the Kockatea Shale, the pore pressure was assumed to be hydrostatic (18.7 MPa). Vertical stress (*S*_*v*_) was estimated to be 47.6 MPa from density logs, minimum horizontal stress (S_hmin_) was 30.1 MPa from leak off tests and the maximum horizontal stress (S_Hmax_) ranged between 45.8 and 50.8 MPa using rock strength and breakout data (Mullen et al. 2018). For fault rock with no cohesion and a coefficient of friction of 0.6, the stress state was found to be transitional strike slip/normal.

If there is a pre‐existing fault plane, frictional sliding will occur when the ratio of the shear stress to effective normal stress is equal to the coefficient of friction of the rock (Jaeger and Cook 1979). For a typical value of *μ* being 0.6 and assuming no cohesion (Zoback and Healy 1984), then the limit can be constrained by:

(4) $$\left(\text{S1}-P_{p}\right)/\left(\text{S3}-P_{p}\right)\leq 3.1$$

where P_*p*_ is the pore pressure, S1 is the maximum principal stress, and S3 is the minimum principal stress (Jaeger and Cook 1979).

Based on Equation 4, for the normal stress case, the ΔP_*p*_ is 3.3 MPa for fault slip. For the strike slip case, the ΔP_*p*_ is 1.8 MPa. The pressure required to create new fractures in the Kockatea Shale at 1910 m in the Woodada 4 well is estimated to vary between 10.7 to 13.2 MPa based on leak off tests conducted in the basal Kockatea Shale at the Woodada 6 well (15.4 MPa/km) and ELL1 well (16.7 MPa/km).

## Sensitivity Analysis

Ten failure envelope scenarios are considered in stereonets for the Kockatea Shale. For the uncemented phyllosilicate case, cohesion is varied between 0 and 0.5 MPa. The coefficient of friction is 0.6 for both cases. The cemented phyllosilicate or healed fault rock scenario is evaluated for the strength estimates at and above breakouts assuming a constant stress gradient. In all cases, stress is varied between strike‐slip and normal. The maximum horizontal stress was constrained by assuming that cohesionless faults exist within the study area (Mullen et al. 2018). This means that while the ΔP_*p*_ estimate is absolute for the cohesionless case, the ΔP_*p*_ for the healed case provides only a relative risk for the purpose of illustrating the sensitivity of the FAS to rock strength. Given the burial history of Woodada and the potential for embrittlement of the Kockatea Shale, a cohesive scenario is justified. The impact of poro‐elasticity in the Kockatea Shale is considered in the Supporting Information. There is a qualitative discussion pertaining to rock strength and poro‐elasticity of the Woodada Formation in the Supporting Information.

In summary, based on the preceding discussion, the vertical stratification of condensate in aquifers and the absence of condensate during logging of some wells supports the proposition that condensate is not as diffusive as methane. The distribution of condensate in aquifers is hypothesized to localize in the vicinity of critically stressed faults as defined by the FAST. Confirmation will validate the minimum fracture stability estimate for fault slip for critically stressed faults associated with leakage of hydrocarbons in the FAST domain.

## Results

**Figure 8:**
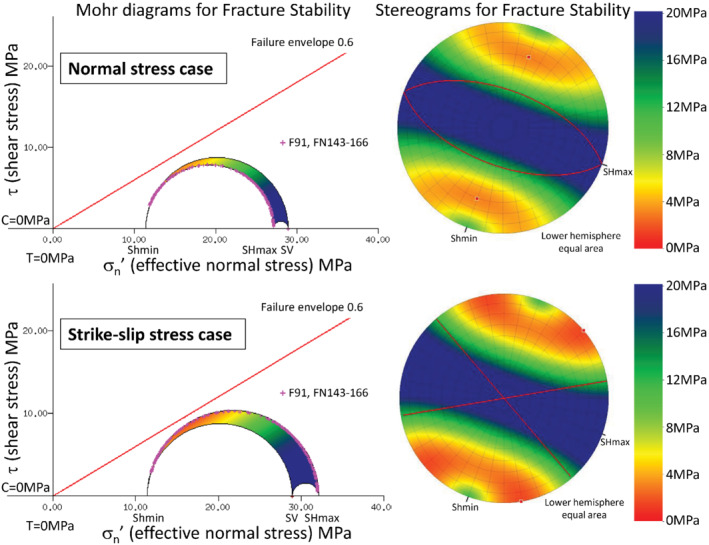
Stereonets for the uncemented case (cohesion of 0 and coefficient of friction of 0.6). The depth was 1910 m, pore pressure 18.7 MPa, S_v_ 47.6 MPa, and S_hmin_ 30.1 MPa. Fracture networks are shown in pink on the Mohr circles. The stereonet for the normal stress case is shown to the top (S_Hmax_ 45.8 MPa). The poles to faults optimally oriented for failure are shown as red dots at 20° and 200°. Faults oriented north‐west (parallel with S_Hmax_) and with dips between 50° and 70° have a ΔP_*p*_ ≤ 4 MPa. Faults with 60° dip (red lines) are optimally oriented for failure, with the lowest ΔP_*p*_ of 3 MPa. The stereonet for the strike slip stress case is shown to the bottom (S_Hmax_ 50.8 MPa.) The poles are at 50° and 170°. The corresponding planes are vertical, striking at 140° and 260°. Faults oriented north‐west and west‐south‐west have a ΔP_*p*_ ≤ 4 MPa for faults with dip 60° to 90°. The lowest ΔP_*p*_ is for vertical faults at 1.6 MPa.

The risk of reactivation of any pre‐existing fault orientation is presented in stereonets. All fault orientations are reported relative to north position. Given that all critically stressed faults are not necessarily leak prone the fracture stability from the FAST is validated by comparison with the pattern of condensate leakage into aquifers. For the normal stress case, uncemented faults oriented west‐north‐west (parallel with S_Hmax_) with dips between 51° to 69° have a fracture stability ΔP_*p*_ of ≤4 MPa. Faults with dips of 60° have the lowest ΔP_*p*_ of 3 MPa (Figure 8). Increasing cohesion from 0 to 0.5 MPa increases the minimum ΔP_*p*_ to 4 MPa (data not shown). For the strike slip stress case, uncemented faults oriented north‐west and west south‐west have a ΔP_*p*_ of 1.7 MPa for faults with dip 40° to 90° (Figure 8). Increasing cohesion to 0.5 increases the ΔP_*p*_ to 2 MPa (data not shown). Overall, fracture stability is greater for the normal stress case.

**Figure 9:**
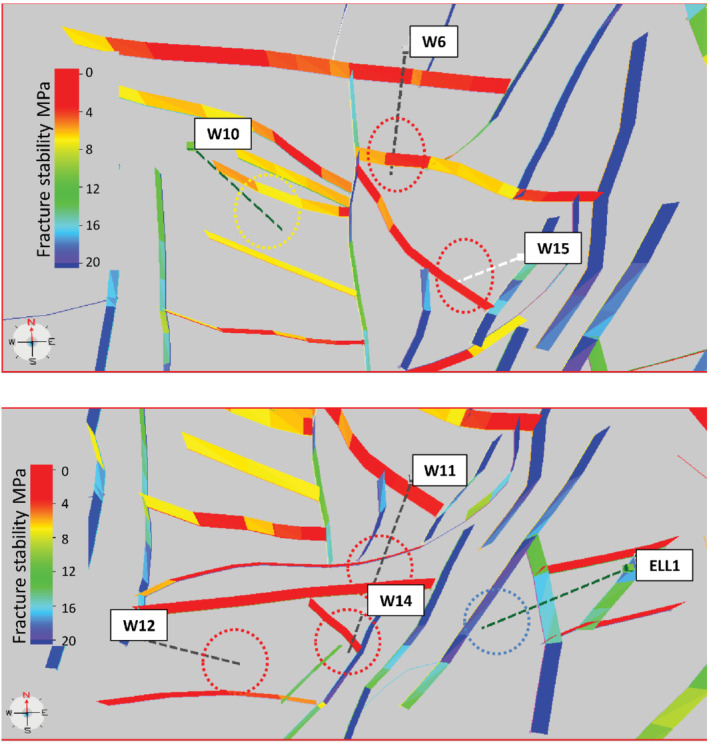
The T7‐TrapTester (Badleys 2016) FAST map is based on a three‐dimensional assessment. The fracture stability scale ranges from 0 to 20 MPa, colored red to blue, respectively. The 280 m radii in plan view in Figure 1 were superimposed on the FAST (dotted circles) for the purpose of assessing the ΔP_*p*_ of the closest fault to each well. Fault planes are rotated to enable visualization of the fault colors. This distorts the image slightly. Parameters are as follows: uncemented rock, fault dip 80° stress parameters as per Figure 8 (strike slip). W = Woodada well. Faults that are critically oriented for shear reactivation (ΔP_*p*_ ≤ 5 MPa) are closest to Woodada 6, 11, 12, 14, and 15 wells (colored red to orange). Faults with high integrity (ΔP_*p*_ > 5 MPa) are closest to Woodada 10 and ELL1 wells colored yellow and blue, respectively.

The FAST map is produced by combining the reactivation risk stereonet with the fault geometry (Figure 9). The FAST map is limited to the strike slip case (worst case for Permian Faults) for uncemented phyllosilicate rock (worst case) with faults dipping 80° (usual case for Permian faults). Faults are a color‐shaded using the same scale as was used for the stereonets.

While methane appears to be relatively mobile in aquifers across the Woodada anticline, the FAST suggests that in aquifers, condensate is localized near critically stressed Permian faults. Wells adjacent to orange‐red faults (low ΔP_*p*_) logged methane and condensate in aquifers and include Woodada 6, 11, 12, and Woodada 14 wells. Wells adjacent to yellow and blue faults (high ΔP_*p*_) logged methane only and include Woodada 10, ELL1 wells, and ELL2 wells (ELL2 is not shown due to its close proximity to ELL1 well).

**Figure 10:**
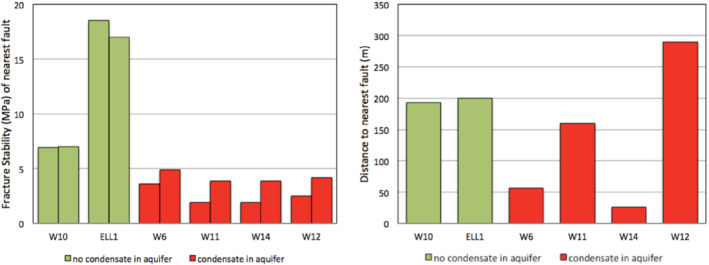
The bar chart on the left shows fracture stability (ΔP_*p*_) by well (strike slip stress in the left bar and normal stress in the right bar). Green are “no condensate” wells and these have a ΔP_*p*_ > 5 MPa. Red are the “condensate wells” and these have a ΔP_*p*_ ≤ 5 MPa. The bar chart on the right is distance to the closest fault for each well (same color code). In this case, there is no correlation between distance and the type of hydrocarbon logged.

The hypothesis that condensate localizes around critically stressed faults due to its limited diffusivity is supported. The FAST map shows that wells that logged condensate in aquifers are proximal to critically stressed faults with ΔP_*p*_ ≤5 MPa. This means relatively small pressure perturbations in critically stressed faults are sufficient to cause hydrocarbon leakage. Wells which did not log condensate are proximal to high integrity faults ΔP_*p*_ >5 MPa (Table 2). This means that even if the pressure increases by 5 MPa, those faults will not leak hydrocarbons. Proximity of wells to faults in itself is not necessarily a risk for an increased probability of detecting condensate in aquifers. For wells that did not log condensate in aquifers, the distance to the nearest fault was 193 to 200 m. For wells which logged condensate in aquifers, the distance to the nearest faults ranged 26 m for Woodada 14 well to 290 m for Woodada 12 well (Table 2 and Figure 10).

**Table 2:** Relationship Between the Distance Between Wells to the Nearest Permian Fault, ΔP_*p*_ and the Presence of Condensate for the Normal and Strike Slip Case Uncemented Phyllosilicate Rock (Parameters as Per Figure 8)

Well	Distance to Nearest Fault (m)	Condensate In Aquifers	ΔP_*p*_ (MPa) Strike Slip	ΔP_*p*_ (MPa) Normal
W10	193	No	6.9	7.0
ELL1	200	No	18.5	17.0
W6	56	Yes	3.6	4.9
W11	160	Yes	1.9	3.9
W14	26	Yes	1.9	3.9
W12	290	Yes	2.5	4.2

On the Woodada anticline, the Woodada 15 well was the only anomaly as hydrocarbons were absent in aquifers based on hot wire detectors. The well intercepted a Permian fault with a ΔP_*p*_ ranging from 3.6 and 5.0 MPa for the strike slip and normal case, respectively. At this location, the Kockatea Shale and Carynginia Limestone were also depleted of gas and this was atypical for the Woodada gasfield. The thickness of the Carynginia Limestone was 94 m and there was no structural explanation for the absence of hydrocarbons .

Stereonets for cemented faults have high integrity (ΔP_*p*_ 14 MPa) for both the strike slip and the normal faulting case (data not shown). For the normal stress case, this includes faults oriented at 110° (parallel with S_Hmax_) and 60° dip. For the strike slip stress case, faults oriented north‐west and west south‐west with moderate to steep dip have the lowest fracture stability. Using the log derived rock strength for rock above the breakouts (20 MPa, coefficient of internal friction of 0.6 and cohesion of 5.8), then the minimum ΔP_*p*_ decreases to 13 and 11 MPa for the normal and strike slip case, respectively.

## Discussion

The Perth Basin is a highly deformed rift basin and the integrity of fault seals cannot be assumed by comparison with unconventional plays in the United States with very different structural histories. The assessment of fault integrity is relevant in the context of hydraulic fracturing in the North Perth Basin. Methane and condensate were logged in aquifers across the Woodada gasfield during drilling of conventional wells based on numeric records and commentary in well completion reports. The only well with a confirmed integrity issue during the production cycle of the gasfield was the Woodada 5 well and the distribution of gas in aquifers is not easily attributed to a leak from this well.

Crest trap faults which are optimally oriented for failure in a strike slip stress regime were suggested to be the likely cause for seal breach in the onshore Kockatea Shale (Mullen et al. 2018). Evidence was presented that suggests the Kockatea Shale may include over pressured intervals across the Woodada anticline. Pressure perturbations in the Kockatea Shale may be sufficient to cause fault slip enabling gas to move vertically into the overlying permeable aquifers. The movement of hydrocarbons once they reach the Lesueur Aquifer is assumed to be controlled primarily by buoyancy and matrix flow father than being restricted to faults consequently both vertical and lateral movement is possible.

Our goal was to test the hypothesis that faults in the Kockatea Shale that are being reactivated in the current stress field are leak prone. To investigate the question of how faults in the Kockatea Shale might affect the movement of gas into aquifers, previously published stress data were used to resolve the shear and effective normal stress acting on individual fault planes across the Woodada anticline. Sensitivity analysis was conducted to determine how fracture stability varied with changes in fault dip, fault orientation, rock strength, and poro‐elasticity.

Based on the FAST analysis, cohesionless normal stress faults oriented west‐north‐west with dips of 60° have the lowest fracture stability (ΔP_*p*_) of 3 MPa. The ΔP_*p*_ increases to 4 MPa for dips of 51° and 69°. While the small ΔP_*p*_ infers a high likelihood of reactivation, most Permian faults have dips between 70° and 80°. However, Jurassic faults which extend into the Kockatea Shale have reduced dips compared with Permian faults (Figure 2) and are not included in the assessment, consequently, the risk may be underestimated. For the strike slip stress case, faults oriented north‐west and west south‐west dipping between 40° and 90° degrees have the lowest ΔP_*p*_ of 2 MPa. The increased range of fault dips with low fracture stability indicates that the risk of fault slip increases with a small increase in the S_Hmax_.

The inputs for the FAST analysis are predicated on the assumption that the Kockatea Shale is a weak shale with a large proportion of clay. For both the strike slip and normal stress case, increasing cohesion from 0 MPa to a more realistic 0.5 MPa had little impact on the absolute value of ΔP_*p*_. A ΔP_*p*_ of 5 MPa was chosen as the critical cutoff for leakage of hydrocarbons from the Kockatea Shale into aquifers via faults for the uncemented normal and strike slip case. This is validated by correlation with the condensate distribution in aquifers over the Woodada anticline.

If the Kockatea Shale is assumed to be a cemented fault rock, the ΔP_*p*_ increases to 14 MPa for both the normal and strike slip cases. In this case, overpressure in the Kockatea Shale is unlikely to be sufficient for fault slip to occur. For a reduced strength in the zone above breakouts, the ΔP_*p*_ decreases to 13 and 11 MPa for the normal and strike slip case, respectively. Despite the relatively higher fracture stability for cemented faults, strain partitioning at the intersections of Jurassic and Permian faults can be associated with an increase in structural permeability based on modeling in the offshore Kockatea Shale (Langhi et al. 2012). Evidence of comparable intersections has been presented for the Woodada anticline (Figure 2). Cemented phyllosilicate fault rocks have the potential to develop highly connected tensile and mixed mode fractures which are more permeable compared with shear fractures alone (Dewhurst and Jones 2003).

The ΔP_*p*_ required to create new fractures in the Kockatea Shale is estimated to vary between 10.7 to 13.2 MPa at 1910 m depth based on leak off tests conducted in the basal Kockatea Shale in the Woodada gasfield . This exceeds the critical ΔP_*p*_ for uncemented faults (<5 MPa), however, is less than for cemented faults (<14 MPa). Leak off tests may not be representative of the pressures required to fracture unconventional targets due to strength anisotropy in shale (Josh et al. 2012). Significantly, in some areas, complex geological fabrics may result in tortuous fractures requiring abnormally high treatment pressures (Nelson et al. 2007). Poro‐elastic scenarios are presented in the **Supporting Section**. Given that the poro‐elastic properties of rocks are poorly understood, if regulators are to specify the maximum ΔP_*p*_ for injection without causing reactivation, then the base case is recommended (ΔP_*p*_ < 5 MPa).

The anomalous absence of hydrocarbons in both the source rock and aquifers at the Woodada 15 well may be related to the fact that the relatively long fault near this well straddles the anticline in a north westerly direction. Fault strike length is a significant factor controlling strain partitioning as reactivation strain tends to progressively localize onto the largest faults over time, which then act to protect neighboring structures (Langhi et al. 2012). There may be increased linkage with unknown Jurassic faults in this area and an associated increased risk of structural permeability.

The Carynginia Shale was hydraulically fractured in 2012 at Woodada 4 well now renamed Woodada Deep (Australian Worldwide Exploration unpublished data). The average UCS of the Carynginia Shale is 34.7 MPa based on standard triaxial testing (Minaeian 2014). The impact of hydraulic fracturing of the Carynginia Shale on potentially weaker overlying formations requires consideration. The stress distribution commonly varies with the elastic properties of different formations and reactivation risk could change depending on how variable these properties are between formations.

The use of gas composition data acquired routinely during the drilling of new wells has several potential applications. Most importantly this kind of data can be used to validate the FAST estimates. This is significant as validated fracture stability estimates can be used to guide regulators in the determination of maximum allowable hydraulic fracturing pressures in environmentally sensitive areas. The gas data also provides insight into the mobility of hydrocarbons in aquifers. For example, the data suggests that hydrocarbons appear to be bypassing the Woodada Formation and the Lesueur Aquifer is functioning as a gas collector zone. The vertical and lateral changes in hydrocarbon concentrations obtained from gas logging can be used to provide indirect evidence of spatial changes in the types of biodegradation processes that may be occurring in the subsurface.

This study is limited in terms of the absence of laboratory‐derived rock strength. In addition, gas reporting is based on numeric records for Woodada 6, 14, and ELL1 wells whereas gas reporting is based on discussion in the well completion reports for ELL2, Woodada 10, 11, and Woodada 12 wells.

## Conclusion

Gas and condensate are naturally occurring in aquifers overlying a conventional gasfield whose source rock is a potential shale gas target. Hydrocarbons were found in dynamic low salinity as well as stagnant saline aquifers suggesting suitably oriented faults are actively functioning as conduits. Fault seal analysis was used to quantitatively assess the stability of faults across the Woodada Anticline. The orientation, dip, and geomechanical properties of the faults and their respective stress states determine whether there is likely to be leakage of gas or of condensate. If faults within the Kockatea Shale are assumed to be comprised of uncemented phyllosilicate fault rock, a ΔP_*p*_ of ≤5 MPa is sufficient to result in hydrocarbon leakage based on the distribution of condensate in aquifers across the Woodada anticline. However, laboratory rock strength data are required to confirm whether the failure envelope inputs are representative of the Kockatea Shale in this area. For cemented fault rock, it is suggested that other leak mechanisms are at play, specifically, Jurassic‐Permian fault intersections. The pressures used in hydraulic fracture stimulation are likely to reactivate faults and lead to further hydrocarbon leakage.

## Authors' Note

The author(s) does not have any conflicts of interest.

## Supporting information


**Table S1:** Source of gas data for the Woodada gasfield.
**Figure S1:** Stereonets for the poro‐elastic uncemented case
