# Supplementary material for: Fault Seal Analysis in an Onshore Unconventional Gas Target, North Perth Basin
Source: Ground Water. 2020 Jul 23;59(1):131–45. doi: 10.1111/gwat.13026 (PMC7891673; doi:10.1111/gwat.13026)
Supplement: Supplementary file 1 — Table S1: Source of gas data for the Woodada gasfield. Figure S1: Stereonets for the poro‐elastic uncemented case [file GWAT-59-131-s001.docx]

**Case study\**

**Fault seal analysis in an onshore unconventional gas target, North Perth Basin**

**Authors: Mullen. F.*, Archer R., Yielding G., Boogaerdt H.**

**Abstract**

During the 1980’s, hydrocarbons were logged in aquifers during drilling of conventional gas wells in the Woodada gasfield. The gasfield is located in the North Perth Basin in Western Australia. Using Fault Seal Analysis Technology, our goal was to test the hypothesis that faults in the Kockatea Shale that are currently being reactivated may be leak prone. Wells proximal to faults with ΔP_p_ > 5MPa logged only methane. Wells proximal to faults with fracture stability (ΔPp) ≤ 5MPa logged both methane and condensate in aquifers confirming that condensate leakage is correlated with critically stressed faults. This assessment assumes that fault rocks in the Kockatea Shale, which is a regional source rock and seal, comprises uncemented phyllosilicate rock. For the normal stress case, faults oriented west-north-west with moderate dip have the lowest integrity. For the strike slip stress case, faults oriented north-west and west-south-west, with moderate to steep dip have the lowest integrity. If the Kockatea Shale fault rock is assumed to be a cemented phyllosilicate then the ΔP_p_ increases to 14MPa for both the normal and strike slip case. In this case, Jurassic-Permian fault intersections may be contributing to hydrocarbon leakage, however, this would require numerical modelling for confirmation. Based on leak off tests, the increase in pressure required to hydraulically fracture the formation varies between 10.7 to 13.8MPa. The treatment pressures used during hydraulic fracturing may potentially exacerbate leakage in areas such as the Woodada gasfield.

**Table S1: Source of gas data for Woodada gasfield.**

| **Well** | **Numeric record** | **Methane** | **Condensate** | **Author** |
| --- | --- | --- | --- | --- |
| Woodada 1 | No | - | - | Hughes &Hughes 1980 |
| Woodada 2 | No | - | - | Hughes &Hughes 1980 |
| Woodada 3 | No | Yes | No | Hughes &Hughes 1981 |
| Woodada 4 | No | - | - | Hughes &Hughes 1981 |
| Woodada 5 | Yes | Yes | Yes | Hudbay Oil 1982 |
| Woodada 6 | Yes | Yes | Yes | Hudbay Oil 1982 |
| Indoon | Yes | Yes | No | Hudbay Oil 1982 |
| ELL1 | Yes | Yes | No | Hudbay Oil 1983 |
| Woodada 8 | No | Yes | No | Strata Oil 1984 |
| Woodada 9 | No | Yes | No | Strata Oil 1984 |
| Woodada 10 | No | Yes | No | Strata Oil 1984 |
| Woodada 11 | No | Yes | Yes | Consolidated Gas 1991 |
| Woodada 12 | No | Yes | Yes | Consolidated gas 1991 |
| ELL2 | No | Yes | No | Consolidated Gas 1992 |
| Woodada 14 | Yes | Yes | Yes | Consolidated gas 1995 |
| Woodada 15 | Yes | No | No | Consolidated Oil 1995 |
| Woodada 16 | Yes | * |  | Phoenix Energy 2001 |
| Woodada 19 | Yes | * |  | Hardman Resources 2019 |

*mud logging started in the Kockatea Shale

**Poro-elastic effects**

Rocks with fluid-filled pores can exhibit poro-elasticity, where changes in pore pressure causes changes in the horizontal principal stresses. The impact of poro-elasticity is assessed to find the coupled stress state which just meets the failure line. This provides an alternate ΔP_p_ which represents the most extreme application of poro-elastic coupling.

In a changing poro-elastic reservoir, S_hmin_ and S_Hmax_ are coupled to the change in pore pressure. The coupling is dependent on Poisson's Ratio (typically 0.25) and the biot coefficient (α) which tends to zero for a crystalline rock with no pores, and 1 for soft saturated soils. For the extreme case of α =1, then S_hmin_ and S_Hmax_ change at about 2/3 of the rate of change of pore-pressure (Zoback 2007). Fracture stability in the presence of a strong poro-elastic effect is assessed by incrementally increasing the horizontal stresses by 2/3 of the increase in pore pressure to the point of failure.

For the end-member poro-elastic case, S_Hmax_ and S_hmin_ are considered to increase at 2/3 of the rate of pore pressure increase until failure occurs. The normal stress case becomes strike slip due to the increase in effective S_Hmax_ to a value larger than the effective S_V_ (Figure 1). The minimum ΔP_p_ increased from a minimum of 3MPa (with no poro-elasticity) to a minimum of 12MPa with poro-elasticity. For the strike slip case, the minimum ΔP_p_ increased from 1.6MPa (with no poro-elasticity) to 4.9MPa with poro-elasticity. Whilst this provides some indication of the impact of poro-elasticity in terms of reducing the probability of failure, the assessment is based on an assumed Biot coefficient of 1 which represents an end-member scenario.


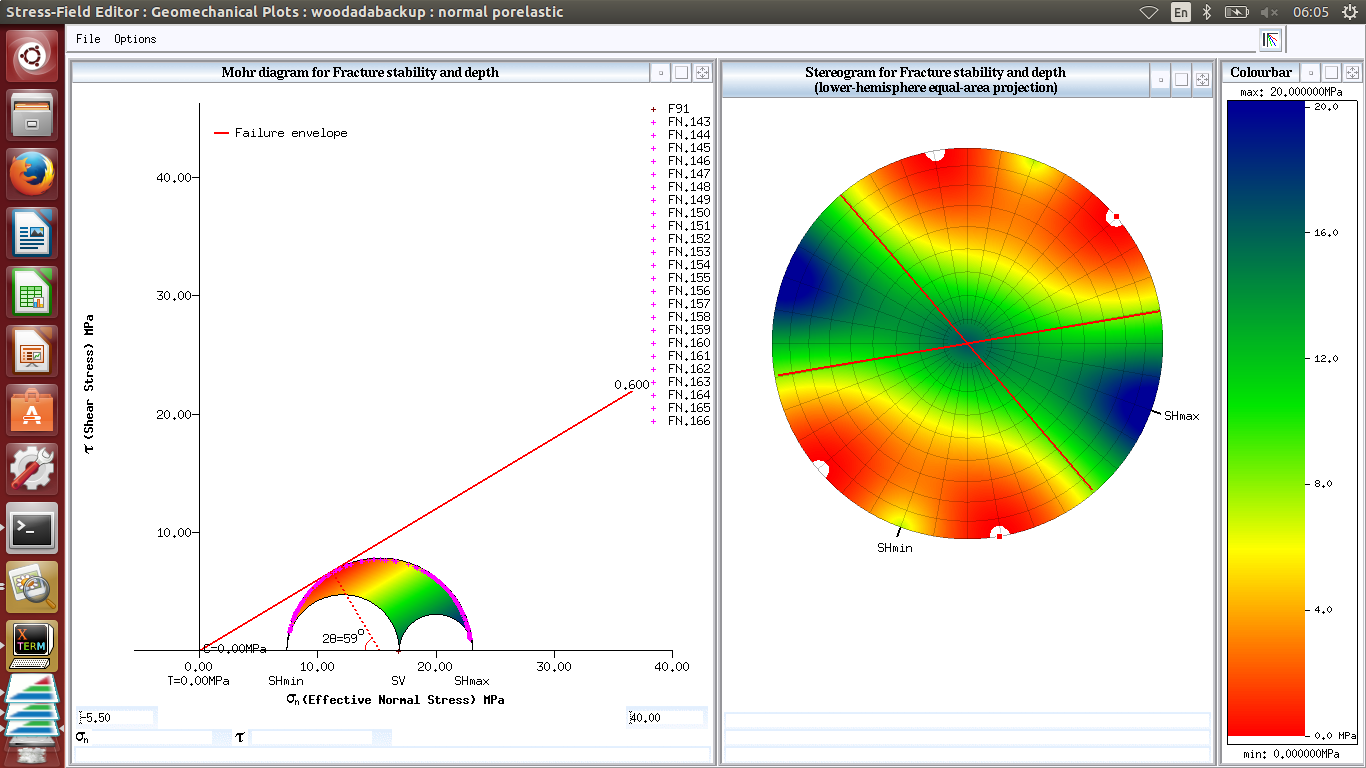


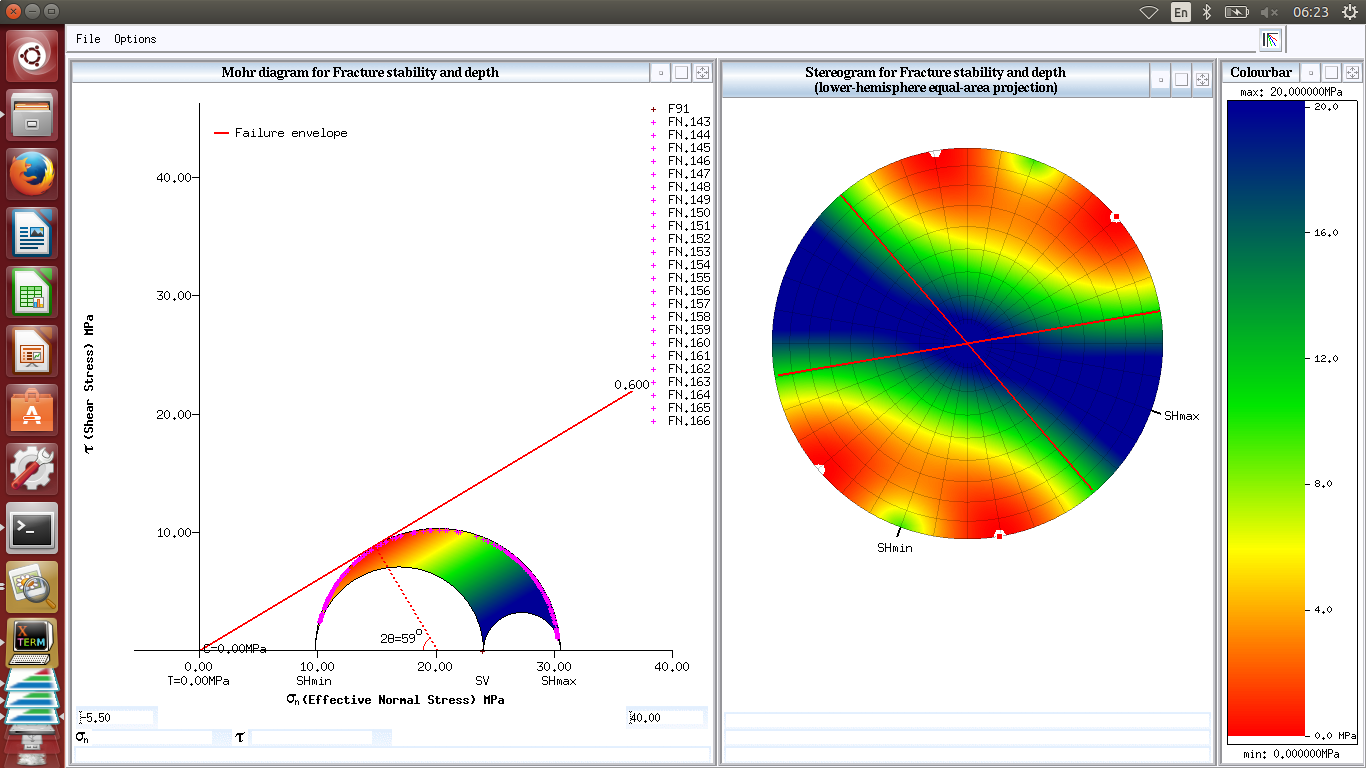


**Figure S1: Stereonets for the poro-elastic case assuming an increase in effective horizontal stress 2/3 of the increase in pore pressure. Sv 47.6MPa. Cohesion of 0 MPa and coefficient of friction of 0.6. The depth is 1910m. The poles are shown as red dots at 50 ̊ and 170 ̊. The corresponding planes are the red lines at 140˚ and 260˚. Fracture networks are shown in pink on the Mohr circles. The normal stress case is shown at the top. The minimum ΔPp increases to 12MPa. The increase for both horizontal stresses is 8MPa with an Shmin of 38.1MPa and an SHmax of 53.8MPa. Poro elasticity results in the normal stress state becoming strike slip. Vertical faults oriented north-west and west south-west have a ΔPp of 0MPa increasing to 6MPa at 40 dip ̊. The stereonet for the strike slip case is shown at the bottom. The minimum ΔPp increases to 4.9MPa with the incorporation of poro-elastic effects. The increase for both horizontal stresses is 3.3MPa (Shmin 33.4MPa and SHmax 54.1MPa). Faults oriented north-west and west south-west have a ΔPp of 0MPa at 90 ̊ increasing to 8MPa at 40 ̊ dip.**

In summary, poro-elastic effects were approximated by increasing the horizontal stress by 2/3 of the pore pressure to a point where failure was reached. The minimum ΔP_p_ increased by a factor of four for the normal stress case (3 to 12MPa) and more than doubled for the strike slip case (2 to 5MPa) for an assumed Biot coefficient of 1.

**Woodada Formation**

Whilst the FAST is only conducted for the Kockatea Shale, the impact of variation of rock strength and poro-elasticity are considered in a qualitative sense for Woodada Formation. The decreasing trend in strength at shallower depths in the Kockatea Shale suggests rock strength may decrease further in the Woodada Formation however, there is no laboratory core data to confirm this. A decrease in strength will reduce the ΔP_p_ assuming a constant stress gradient with depth. The empirical formula used to assess strength in the Kockatea Shale is suitable for low porosity shale and cannot be used for the Woodada Formation which is permeable to water.

Poro elastic effects may be more pronounced in the relatively more permeable faults in Woodada Formation resulting in an increase in the fracture stability. However, if faults in the Kockatea Shale are contributing to gas leakage, then the occurrence of gas in the Woodada Formation suggests that the poro-elastic properties of faults in the aquifer are insufficient to stop gas leakage.

Water influx was noted during drilling the Woodada 14 well at rates of 1Kilolitre/min at 1514m where the well crossed a fault in the Woodada Formation (Consolidated Gas 1994) suggesting the faults are water saturated and permeable. In permeable formations, matrix flow dominates and the significance of fracture stability as a mechanism for creating permeability is reduced. The impact of an increase in water saturation on gas flow in faults crossing into the Woodada Formation is beyond the scope of FAST. Multiphase flow modelling can account for changes in relative permeability of gas in water saturated faults in the Woodada Formation.

Disclaimer: Please note ‘Supporting Information’ is generally not peer reviewed.
